# Biopolymers for Tissue Engineering: Crosslinking, Printing Techniques, and Applications

**DOI:** 10.3390/gels9110890

**Published:** 2023-11-10

**Authors:** David Patrocinio, Victor Galván-Chacón, J. Carlos Gómez-Blanco, Sonia P. Miguel, Jorge Loureiro, Maximiano P. Ribeiro, Paula Coutinho, J. Blas Pagador, Francisco M. Sanchez-Margallo

**Affiliations:** 1 CCMIJU, Bioengineering and Health Technologies, Jesus Usón Minimally Invasive Surgery Center, 10071 Cáceres, Spain; dpatrocinio@ccmijesususon.com (D.P.); vpgalvan@ccmijesususon.com (V.G.-C.); jbpagador@ccmijesususon.com (J.B.P.); 2 CPIRN-IPG, Center of Potential and Innovation of Natural Resources, Polytechnic of Guarda, 6300-559 Guarda, Portugalmribeiro@ipg.pt (M.P.R.); 3 CICS-UBI, Health Science Research Center, University of Beira Interior, 6201-506 Covilhã, Portugal; 4 CIBER CV, Centro de Investigación Biomédica en Red—Enfermedades Cardiovasculares, 28029 Madrid, Spain; msanchez@ccmijesususon.com; 5 Scientific Direction, Jesus Usón Minimally Invasive Surgery Center, 10071 Cáceres, Spain; 6 TERAV/ISCIII, Red Española de Terapias Avanzadas, Instituto de Salud Carlos III (RICORS, RD21/0017/0029), 28029 Madrid, Spain

**Keywords:** natural polymers, hydrogels, crosslinking, bioprinting techniques, tissue engineering

## Abstract

Currently, tissue engineering has been dedicated to the development of 3D structures through bioprinting techniques that aim to obtain personalized, dynamic, and complex hydrogel 3D structures. Among the different materials used for the fabrication of such structures, proteins and polysaccharides are the main biological compounds (biopolymers) selected for the bioink formulation. These biomaterials obtained from natural sources are commonly compatible with tissues and cells (biocompatibility), friendly with biological digestion processes (biodegradability), and provide specific macromolecular structural and mechanical properties (biomimicry). However, the rheological behaviors of these natural-based bioinks constitute the main challenge of the cell-laden printing process (bioprinting). For this reason, bioprinting usually requires chemical modifications and/or inter-macromolecular crosslinking. In this sense, a comprehensive analysis describing these biopolymers (natural proteins and polysaccharides)-based bioinks, their modifications, and their stimuli-responsive nature is performed. This manuscript is organized into three sections: (1) tissue engineering application, (2) crosslinking, and (3) bioprinting techniques, analyzing the current challenges and strengths of biopolymers in bioprinting. In conclusion, all hydrogels try to resemble extracellular matrix properties for bioprinted structures while maintaining good printability and stability during the printing process.

## 1. Introduction

Tissue engineering (TE), as it is currently known, is a relatively recent field of innovation resulting from a complex merger of pre-existing lines of work from three different areas of knowledge: engineering, clinical medicine, and the biomedical field [1]. The TE area emerges as a consequence of clinical challenges related to the unavailability of tissue samples for the replacement of a damaged area when an accident or injury occurs and the immunological response associated with allograft transplants. In addition, autografts and allograft-based therapeutic approaches are associated with low availability, significant morbidity, and the requirement of immunosuppressive drugs for long-term treatment [2,3]. Driven by the pressing clinical need to develop alternative treatment therapies that can achieve the same outcomes as autografts and allografts but without their associated drawbacks, the field of tissue engineering emerged and has seen rapid progress over the past few decades [1].

In this way, TE aims to restore, maintain, improve, or replace different types of tissues and ultimately to incorporate or integrate the in vitro formed tissue into the body, contributing to the repair of injuries or the replacement of failing organs [1]. To accomplish these goals, TE proposes the production of provisional structures with structural and functional features that mimic the native tissue. In this field, different bioengineered constructs have been developed by employing different techniques, such as matrix decellularization [2,3], injectable hydrogels [4,5], and bioprinting [6,7]. In the first stage, the TE goal was to produce tissue constructs using biological or engineering techniques, but the evolution in this area has been contributing to the exploration of more integrated strategies involving the use of a biomaterial or biomimetic platform in combination with cells and biological factors that prompt the tissue regeneration process [6].

For the production of such biomimetic 3D supports, bioprinting is a promising technology that has revolutionized the TE and regenerative medicine fields, among others. Through this technology, it is possible to produce tissue models in a high-throughput manner, creating porous structures with controlled architecture using different biomaterials in combination with viable and physiologically relevant cells. Bioprinting enables the achievement of appropriate cell distribution, attachment, and growth within the biomaterial scaffold, which is complemented by improved nutrient and gas exchange as well as enhanced cell-cell communication due to the interconnected pores and large surface area [8,9]. In this way, bioprinting can contribute to overcoming the lack of dynamic and complex tissue structure in 2D cell culture as well as provide 3D scaffolds with spatial depth and more realistic cell-cell communication to better understand and simulate in vivo physiology [9]. Furthermore, the efficiency of 3D bioprinters and the availability of bioinks (polymer solutions and viable cells) with suitable structural, physicochemical, mechanical, thermal, and biological features are key factors for the success of bioprinting technology [9]. To be considered suitable for bioprinting, bioinks must have proper mechanical, rheological, chemical, and biological attributes [10]. This means that the materials used for the bioink formulation must ensure their printability, adequate mechanical properties according to the targeted tissue, high resolution and shape fidelity if needed, as well as biocompatibility, biodegradability, bioactivity, and reliability mimicking the microenvironment of tissues [11]. Thus, the choice of (bio)printable materials continues to be the bottleneck in bioprinting technology since it is required to use a polymer solution with suitable rheological and biological properties for bioprinting technique and cell incorporation, respectively. In addition, the printing techniques, such as photopolymerization, laser, extrusion, and droplets are another parameter that can be considered according to the properties of the polymeric bioink.

In this way, hydrogels arise as promising materials for TE and bioprinting, as they are characterized as highly biocompatible materials that allow the incorporation of cells and bioactive compounds. Moreover, their highly porous microstructures allow better cell internalization into the matrix and promote cell proliferation, owing to optimized oxygen and nutrient diffusion [12,13]. Biocompatibility, biodegradability, and specific structural and mechanical properties need to be ensured when developing a hydrogel formulation for TE, and more so when the formulation is intended for bioprinting, as bioprinter-specific requirements need to be considered. It is also important to note that natural materials are more sustainable than synthetic ones because not only are they degradable, but also, in most cases, they are obtained from renewal sources or by-products, such as proteins (e.g., gelatin, sericin), collagens [14], polysaccharides such as chitosan (CS) [15], alginate, or other natural-derived polysaccharides. Many hydrogel formulations contain polymers with hydrophilic groups in their structure, which confers enhanced interactions with biological tissues [16]. Similarly, a swollen-state hydrogel has a low interfacial contact angle with biological fluids, which decreases the chances of a negative immune response, leading to excessive inflammation, interference with healing, and implant rejection [17,18,19]. Another interesting characteristic of hydrogels is their stimuli-responsive nature due to their capacity to perform structural or mechanical changes in response to environmental signals. The stimuli-responsive nature can be classified into (i) physical, (ii) chemical, and (iii) biological, which allows the formulation of tunable bioinks for bioprinting [20].

To the best of our knowledge, this review highlights the characteristics and development of biological hydrogel-based bioinks for use in TE applications, describing the methodological modification/functionalization approaches, crosslinking techniques, and their respective applications on TE. Finally, the main challenges and future steps in using natural-based hydrogels in the 3D bioprinting area are also depicted.

## 2. Biopolymers for TE-Formulated Bioinks

Natural polymers are promising materials considering their intrinsic physicochemical and biological properties, mainly due to their large molecular chains and monomeric units. As previously outlined, natural polysaccharides and proteins are widely used in the development of scaffolds in regenerative medicine. Also, in the bioprinting field, biopolymers received special interest for their rheological and tribological features that allow them to generate complex geometries and structures of different tissues, as well as providing support for cell adhesion and proliferation. In this section, recent reports on proteins and polysaccharides used in 3D bioprinted hydrogels for TE are depicted.

### 2.1. Proteins

Collagen (Col) and fibrin (F) are proteins found in the Extracellular Matrix (ECM) of mammals and present specific bio-related features, such as biocompatibility and biodegradability [21], which is why they have been selected as bioinks to produce 3D bioprinted structures. In this way, several authors selected ECM structural proteins to produce their hydrogels and ensure provisional support for cell growth as well as mimicking the microenvironment available on the native tissue [22,23,24,25,26,27,28,29,30]. The rheological properties provided by these proteins are critical for bioprinting because of their weak mechanical behavior, which compromises the printability and consequently the integrity of the bioprinted material.

Among them, proteins such as Col, gelatin (Gel), silk fibroin (SF), and F have been used as bioinks to produce hydrogels for TE, as depicted in the following subsections and summarized in Table 1.

#### 2.1.1. Collagen

As the main structural protein of the ECM, Col has a high affinity for adherent cells [38], and Col hydrogels are widely used in 3D bioprinting purposes due to their good biocompatibility and bioactivity [39,40,41,42]. However, the weak mechanical properties and constraints in sterilization are major challenges [43]. To improve the material properties, a chemical modification can be performed by adding methacrylate (MA) moieties, allowing stronger crosslinking by intermolecular radical reactions [44].

It is important to note that these methacrylate groups can cause two undesirable effects on cells. First, free radical initiators could affect cell membranes under oxidative stress. Secondly, methacrylate and diacrylate chemical groups could provoke immunological responses in cells due to incomplete degradation of the scaffold, resulting in toxicity or disruption of the cell-cell interaction [45,46].

Also, it can be combined with other materials to generate a hydrogel that can be stabilized after printing [47]. In general, bioprinted Col hydrogels are used in microextrusion, inkjet, or laser-assisted bioprinting strategies [48]. So, this natural polymer can be used per se or combined with other compounds and cells to generate bioinks with promising properties for TE applications.

For example, Col was used as a bioink in the development of 3D skin substitutes by Yoon et al. The authors used extrusion bioprinting to construct Col 3D scaffolds seeded with primary human epidermal Gel keratinocytes (HEK) and human dermal fibroblasts (HDF). As the main findings, the results evidenced that the cell-laden 3D scaffolds implanted on a 1 × 1 cm^2^ full-thickness excision mouse model were able to promote complete skin regeneration after one week of implantation [49].

On the other hand, Col combined with fibrinogen, autologous dermal fibroblasts, and epidermal keratinocytes were tested as bioinks on laser in situ bioprinting on murine full-thickness wound models (3 × 2.5 cm) and porcine full-thickness wound models (10 × 10 cm). In general, the results evidenced rapid wound closure, reduced contraction, and accelerated re-epithelialization [50].

In addition, Col was also used as bioink in the production of 3D bioprinted constructs for bone regeneration applications. For example, Kim and Kim combined Col with a bioceramic (β-TCP), preosteoblasts (MC3T3-E1), and human adipose stem cells (hASCs). The cell-laden structure was mechanically stable, and the cells remain viable, resulting in their proliferation and osteogenic differentiation [51].

#### 2.1.2. Gelatin

Gelatin (Gel) is a low-cost biopolymer obtained by partial hydrolysis of Col, possessing a significant amount of arginine-glycine-aspartic acid (RGD), which will prompt cell attachment [52,53]. Furthermore, Gel is water-soluble and biodegradable through the action of enzymes such as collagenases (MMP-1 and MMP-8), having lower antigenicity than Col [54]. Indeed, different studies have evidenced the beneficial effects of Gel on cell adhesion, migration, and growth in the tissue regeneration process [55,56,57].

For bioprinting purposes, the solubility of Gel in warm water and its gelation at low temperatures allow the formation of physical crosslinked hydrogels [35,58]. Also, it is possible to increase the viscosity of bioinks for extrusion-based printing by decreasing the melting temperature of non-modified Gel. However, to obtain more stable scaffolds for TE, chemically crosslinked Gel hydrogels have been explored through the modification of Gel with methacryl groups [59,60].

In 2018, Yin et al. combined methacrylate gelatin with Gel to obtain stable structures through the extrusion-based bioprinting technique. So, the printed structures were mechanically stable, and the cell printing of bone marrow stem cells did not compromise their viability [61].

In addition, a composite biomaterial ink composed of Fish Scale and alginate dialdehyde (ADA)-Gel for the fabrication of 3D cell-laden hydrogels using mouse pre-osteoblast MC3T3-E1 cells was revealed as a suitable biomaterial ink formulation with great potential for 3D bioprinting for bone TE applications [62].

A semi-synthetic bioink composed of 4.0% Gel, 0.75% alginate, and 1.4% carboxymethylated cellulose nanocrystal dissolved in 4.6% D-mannitol with normal human knee articular chondrocytes (NHAC-kn) was formulated and evaluated for 3D bioprinting. The results demonstrated the bioink as printable, stable under cell culture conditions, biocompatible, and able to maintain the native phenotype of chondrocytes, which, aside from meniscal tissue bioprinting, is suggested as a basis for the development of bioinks for various tissues [63].

Moreover, bioink hydrogel based on Gel blended with other polymers, such as phenylboronic acid-grafted hyaluronic acid (HA-PBA) and poly(vinyl alcohol), demonstrates that it can produce 3D bioprinted structures with anti-ROS ability. They try to improve cartilage regeneration in a chronic inflammatory and elevated ROS microenvironment [64].

Also, in Table 1, some recent works reporting the application of bioprinted Gel hydrogels for TE applications are summarized.

#### 2.1.3. Silk Fibroin

Silk fibroin (SF) is extracted from silkworm silk and has been used as a potential biopolymer for TE due to its physicochemical and biological properties such as biocompatibility, biodegradability, low immunogenicity, and tunable mechanical properties [35,65,66]. On the other hand, silk biomaterials have received FDA approval for some medical products, such as sutures and surgical meshes, and can be processed into a variety of material formats [67,68]. Indeed, SF promotes the proliferation and adherence of different cells and avoids inflammation processes [69,70,71]. It can also be combined synergistically with other polymers by chemical interactions or covalent bonds, enabling physical crosslinking and thus avoiding harsh crosslinking chemicals.

An alternative way to use SF as bioink is based on its chemical modification with methacrylate groups that enable its photocrosslinking, resulting in a semi-synthetic SF. In this way, Yang et al. used methacrylated SF and metacrylated gelatin together in a new bioink to obtain rheological properties suitable for extrusion bioprinting. This bioink was able to encapsulate human umbilical vein endothelial and rat pheochromocytoma (PC12) cells, which maintain high viability and proliferation ability after the printing process. Also, the printed structures were implanted in the subcutaneous tissue of rats, revealing their promising potential to be used in TE applications [72].

In turn, a 3D-printed hydrogel based on alginate-silk nanofibril was introduced for soft tissue engineering, in which silk nanofibril reinforced Alg and was responsible for modulating its injectability by improving its shear-thinning behavior and shape retention before ionic crosslinking and supporting cell attachment and proliferation, with suitable physical and mechanical properties for 3D-printed structures in complex shapes such as ear cartilage [73]. Nevertheless, the low viscosity and frequent clogging during printing are some barriers described for SF use as bioink [74,75], despite the reports on the production of diverse 3D bioprinted structures for TE applications as described in Table 1.

#### 2.1.4. Fibrin

Fibrin (F), as well as its precursor fibrinogen, belong to the family of glycoproteins that are crucial to induced blood clotting. Its three-dimensional network composed of randomly arranged fibers prevents blood leakage and contributes to the migration of cells towards the injury site, leading to the tissue regeneration process [76]. For these reasons, F has been widely used in the biomedical field due to its excellent biocompatibility and biodegradability, as well as the easily 3D processable capabilities of fibrin hydrogels [28,77].

Budharaju et al. reported a dual crosslinking strategy utilized towards 3D bioprinting of myocardial constructs by using a combination of alginate and fibrinogen, and it is based on a pre-crosslinking of the physically blended alginate-fibrinogen bioinks with CaCl_2_ for improving shape fidelity and printability. This bioink was revealed to be cytocompatible and possesses the potential to be used for the biofabrication of thick myocardial constructs for regenerative medicine applications [78].

Additionally, other works reported a new droplet-based bioprinting system to integrate a cell-laden hydrogel with a microfibrous mesh produced by embedding human dermal fibroblasts in a collagen-alginate-fibrin hydrogel matrix. This study shows that cell-hydrogel-microfibre composites maintain high cell viability and promote cell–cell and cell–biomaterial interactions, offering an efficient way to create highly functional tissue precursors for laminar tissue engineering, particularly for wound healing and skin tissue engineering applications [79].

However, for bioprinting applications, Fibrin cannot withstand a stable 3D shape due to its low viscosity and Newtonian fluid behavior [80]. Therefore, different strategies have been developed through the combination of F with printable biomaterials, such as Gel and polyethylene glycol (PEG) [81,82], using a support bath to embed the printed structure [22,80], or crosslinking the hydrogel during the printing process [83]. Due to these reasons, the production of bioprinted fibrin-based hydrogels remains a challenge, and there are yet few works available in the literature, as listed in Table 1.

#### 2.1.5. Other Proteins

In addition, some reports addressed the use of decellularized matrix and Matrigel as bioinks for TE purposes. The decellularized matrix (dECM) is obtained through physical and/or chemical methods to remove all cellular components while maintaining the ultrastructure and composition of the ECM [84]. As a bioink, dECM does not require the use of crosslinkers and allows to mimic tissue-specific characteristics into printed constructs [85,86,87], but any additional structural biopolymers are needed to recover its structural stability [84]. In fact, porcine-derived dECM bioink from different sources (liver, heart, skin, and cornea) was tested, and the authors verified that the tissue source of the dECM induced tissue-specific gene expression in human bone marrow mesenchymal stem cells [86]. On the other hand, dECM bioinks have been validated for skin regeneration applications, where a printed pre-vascularized skin patch promoted wound closure, epithelialization, and neovascularization [88]. In other work, the skin-derived dECM combined with a fibrinogen-based bioink incorporated with primary human skin fibroblasts improved the mechanical properties and viability of a bioprinted skin model [89]. Also, dECM as bioink was studied for the printing of human cardiac progenitor cells, demonstrating that the printed pre-vascularized stem cell patches promoted vascularization, maintained cell viability, and decreased cardiac remodeling and fibrosis [90,91].

Similarly, Matrigel consists of a composite, gelatinous mixture that mimics the human ECM and contains proteins such as laminin, Col, and entactin, which prompt cell growth and adhesion [92,93]. As recently reviewed, Matrigel applications in TE and bioprinting are based on the efficiency of cell differentiation, promoting angiogenesis and tissue regeneration both in vitro and in vivo [94].

In a work conducted by Li, the combination of Matrigel with sodium alginate was used to produce a hydrogel that was suitable for the growth of ectomesenchymal stem cells [95]. However, Matrigel has poor mechanical strength and requires a temperature-controlled system when used for extrusion printing [96,97], which limits its application in 3D bioprinting.

### 2.2. Polysaccharides

Among natural polymers, polysaccharides are described as the most promising macromolecules for biomedical applications. The most explored polysaccharides for biomedical applications are alginate, chitosan (CS), hyaluronic acid (HA), or cellulose [98,99], and they provide structural support in different cellular walls. These biopolymers are biosynthesized by living organisms, including plants, animals, algae, bacteria, and fungi [100], and present several important features crucial for 3D bioprinting applications in TE. The remarkable features of polysaccharides are their biocompatibility with mammalian cells and tissues and their biodegradability under physiological conditions, with the formation of nontoxic degradable products [20,101]. Moreover, these biopolymers are classified as eco-friendly materials due to their renewable and biodegradable nature [100], and they are considered safe (GRAS), being widely applied in the pharmaceutical industry as authorized excipients [102]. However, some studies concluded that alginate does not properly interact with mammalian cells, promoting minimal protein adsorption [103]. This feature allows researchers to develop new solutions that can be easily and quickly translated to the clinic. Nevertheless, the new 3D bioprinting technology implies a new challenge for biomedical sciences, namely regarding the development of bioinks able to provide adequate printability and simultaneously the mechanical and biological features needed for the TE field. In turn, the main weakness of biopolymer applications in 3D bioprinting is the difficulty in obtaining scaffolds with reproducible quality and properties and, to some extent, the lack of mechanical properties supporting their application as bioinks [104]. In this context, these polymers have been combined and blended with other natural and synthetic polymers to assure their application as bioinks for 3D bioprinting, as summarized in Table 2.

#### 2.2.1. Alginate

Alginate is a low-cost FDA-approved natural polysaccharide and probably the most used biomaterial in TE because it mimics the functions of the ECM and also has the possibility of being functionalized with cell adhesive links. Moreover, as a bioink, alginate has tunable degradation kinetics and can gelate easily [117,118,119,120].

Alginate solutions present the characteristic behavior of non-Newtonian fluids at low viscosities, which makes the production of a 3D geometrically defined structure difficult. Therefore, effective 3D printing alginate structures usually require crosslinking or the addition of thickening agents aiming to facilitate the solution extrusion as a filament [121]. For this reason, different approaches have been explored, from ionic crosslinking to combinations with other polymers.

Alginate is widely employed in vascular, cartilage, and bone tissue printing, as reviewed by [122,123]. The most successful applications of alginate as bioink are related to cartilage printing and bioprinting of vascularized tissues, for which the employment of coaxial (or triaxial) nozzle assemblies for printing alginate-based bioinks highlights excellent results. However, the mechanical performance of alginate compromises its application in bone tissue engineering, which can be overcome by combining alginate with other biomaterials such as Gel, hydroxyapatite, polycaprolactone, polyphosphate, or biosilica.

#### 2.2.2. Chitosan

Chitosan (CS) is also highly used in TE due to its low price and some critical biological properties, such as biodegradability, biocompatibility, and antimicrobial activity [124]. Indeed, CS supports adequate cell proliferation and differentiation, mimicking the native tissue structure, and provides a suitable microenvironment for oxygen and nutrition exchanges [125,126]. In addition, the presence of CS in the composition of bioinks confers high antimicrobial activity due to the electrostatic interaction of the protonated NH3+ in CS with the negative cell wall of bacteria, which causes bacterial death or restricts their growth [127].

Further, the CS-containing solutions remain stable under physiological conditions, possessing appropriate viscosity values for bioprinting purposes [128,129]. However, as a natural polymer, CS presents a slow gelation rate and a weak mechanical strength [130,131,132]. Such a drawback has been overcome through combination with other polymers or the production of semi-synthetic CS derivatives. For example, the approach involves CS coupling with methacrylic anhydride (methacrylation of the backbone) to promote stronger crosslinking [133]. In Table 2, different CS-based bioinks produced for TE are listed.

In fact, the use of CS as bioink for TE applications has been widely explored, as evidenced in different reviews [134,135,136].

#### 2.2.3. Hyaluronic Acid

Another remarkable polysaccharide is hyaluronic acid (HA), which is a linear polysaccharide with an important role in ECM formation, especially in connective tissues. HA plays key roles in cell proliferation and differentiation, immune modulation, and angiogenesis since it can mediate cell activity through receptor-ligand interactions with surface receptors such as CD44 and RHAMM [137,138]. In addition, HA structure is amenable to chemical modification through the grafting of active moieties or crosslinking, and diverse HA derivatives are available for clinical applications [138,139].

Two main limitations of using HA as a bioink are its limited mechanical performance and its viscous shear-thinning behavior, which hinders the interaction and fusion of printed filaments or droplets, preventing it from maintaining the desired shape throughout the printing process [140,141,142]. Because of this, HA is usually combined with other biomaterials, including natural or synthetic polymers, to obtain 3D bioprinted matrices for tissue regeneration applications, resulting in the creation of stable structures that can ensure cell viability during the extrusion process. In Table 2, the most recent works reporting the development of HA-based 3D bioprinted matrices for TE purposes are depicted.

Indeed, the intrinsic properties of HA make it an ideal choice of bioink for developing tissue constructs. Some reviews available in the literature describe the physicochemical properties, reaction chemistry involved in various cross-linking strategies, and biomedical applications of HA. Further, the features of HA bioinks, emerging strategies in HA bioink preparations, and their applications in 3D bioprinting have also been depicted [142,143].

#### 2.2.4. Cellulose

Cellulose, mainly obtained from plants (Cellulose Nanofibril or CNF), but also produced by some bacteria (e.g., Cellulose Nanocrystals or CNC), is one of the most abundant biomaterials on earth [144] This biomaterial provides a stable matrix for TE with good mechanical properties. Another interesting feature is that inside the human organism, cellulose behaves as a non-degradable or very slowly degradable material [145,146]. Due to these reasons, cellulose is the main biopolymer in medical products, reinforcing its safety for humans.

For 3D bioprinting purposes, cellulose can support different process parameters, while its high viscosity, high surface area, good mechanical stability, tunable surface chemistry, and shear-thinning behavior allow the creation of 3D-printed freestanding and biocompatible constructs with favorable mechanical properties. However, it is necessary to use polymeric combinations or cellulose derivatives to improve the ink printability and shape fidelity after printing [147,148,149]. Nanocelluloses have attracted significant interest in the field of bioprinting, with previous research outlining the value of nanocellulose fibrils and bacterial nanocelluloses for 3D bioprinting tissues such as cartilage, augmenting the printability and chondrogenicity of bioinks [150], and as reviewed by [151], with important features for TE with different applications from vascular prosthesis to neural and bone regeneration. Furthermore, multicomponent bioinks based on pectin (Pc) and TEMPO-oxidized cellulose nanofibers (TOCNFs) for extrusion-based applications are used in complex-shaped scaffolds for tissue engineering [152] and in the production of hybrid hydrogels made of chemically modified pectin, Gel, and xanthan gum to create a supportive environment for cell adhesion and proliferation essential for wound healing and to incorporate cells in 3D bioprinting applications [153].

Moreover, novel, biodegradable, cost-effective, antimicrobial-loaded scaffolds are an emergent trend in biomedical devices and medical equipment manufacturing. So, hydrogel-based bioinks composed of cellulose and derivates revealed significant antibacterial and antibiofilm properties, suggesting the promising potential to be introduced in 3D bioprinting technology [154].

#### 2.2.5. Gellan Gum

As a linear microbial exopolysaccharide, Gellan gum (GG) is a low-cost biopolymer that exhibits a variety of desirable properties, such as biocompatibility and biodegradability. Furthermore, according to their degrees of methylation, GG can show high transparency, thermo-responsive characteristics, flexible mechanical properties, efficient gelling, ease of manufacturing and crosslinking, and stability under physiological conditions that make it a compelling option as a bioink [155,156,157].

GG has been used to create bioprintable hydrogel when physically combined with lignin and crosslinked with magnesium ions, leading to improved in vitro chondrogenesis of hMSCs for cartilage regeneration [158]. Furthermore, when blended with Gel, it was used for vascularized bone regeneration, resulting in a hydrogel scaffold with a controlled release of deferoxamine that significantly promoted angiogenesis and osteogenic differentiation [159], as well as for adipose tissue engineering [155].

A recent review conducted by Cernencu described the various strategies that have been employed to propel the use of GG as bioink [160]. Also, a synopsis of the printable ink designs (e.g., compositions and fabrication approaches) that may be explored for tuning the properties of GG-based 3D-printed hydrogels for TE applications was discussed. Such work outlined the development of GG-based 3D printing inks, highlighting their possible biomedical applications.

#### 2.2.6. Other Polysaccharides

Other polysaccharides of natural origin, such as xanthan, have been proposed as bioinks considering their biological activities. Xanthan gum (XG) is a heteropolysaccharide of high molecular weight produced by Xanthomonas campestris. The rheological properties of XG are attractive, as they enable the formation of pseudoplastic solutions at low concentrations. Additionally, XG exhibits good biocompatibility and is considered safe for various biomedical applications [161]. Moreover, it has been used in 3D bioprinting to produce a multilayered 3D construct by extrusion techniques on a hydrogel-based formulation containing alginate chemically crosslinked with SrCl_2_, which can be conveniently sterilized via steam heat. Furthermore, its good rheological properties make it a cost-effective option to obtain a bioink able to produce biological tissues and other applications [162]. It can also be blended with Gel crosslinked with GTA to obtain a 3D-printed hydrogel that is biocompatible, maintains its print shape, resists swelling, and degrades easily [108]. Moreover, enzymatic polymerization or methacrylation has been proposed for the obtainment of semi-synthetic xanthan gum to modulate its printability [110] and biofunctionality [163].

Additionally, dextran (Dex) is a nontoxic and hydrophilic homopolysaccharide that can be used to produce biodegradable scaffolds due to its degradation by dextranase. As a bioink, dextran is usually combined with other biomaterials to modify its poor mechanical strength [164,165]. So, Dex was used for microextrusion hydrogels with chemically crosslinked Gel-varying ratios of Dex polyaldehyde. This material has the potential to: (1) form self-supporting structures in multiple layers that can be combined with living cells [166], and (2) produce a biocompatible and printable hydrogel using thermosensitive Gel and oxidized dextran with a tunable gelation time that can be easily post-reinforced through Schiff base crosslinking [167]. Dex exhibits remarkable properties in the TE field, specifically in skin and wound regeneration, when combined or modified with other biopolymers.

Starch is a neutral polysaccharide produced by plants such as rice, wheat, or maize. This polysaccharide is insoluble in water at room temperature, but its granules swell and gelatinize when heated. By cooling the starch suspension, the amylose phase separates, promoting the formation of a highly stable and biocompatible gel [168,169]. For bioprinting purposes, starch has been chemically modified or combined with other biomaterials to obtain hydrogels with good mechanical and rheological properties as well as enable the printing of structures without the need for any additional heat treatment. Thus, some authors have proposed starch as a highly desirable bio-ink to promote 3D TE by blending it with Gel nanoparticles and Col [170] or with CS in a 50/50 ratio for neural TE application [171].

Apart from alginates, other macroalgae-based polysaccharides have been evaluated for their suitability to be used in 3D bioprinting. Carrageenan (CA) is widely used in TE for its resemblance to the natural glycosaminoglycans of ECM. Despite the important properties of k-carrageenan (κ-CA) such as biocompatibility, biodegradability, shear-thinning, and ionic gelation, its gelation properties are challenging to control, which limits its use for applications in 3D bioprinting. To overcome this limitation, various modifications to the crosslinking process were tested to enhance the rheological properties of extrusion-based 3D-printed scaffolds. Several studies have reported the use of k-carrageenan-based bioinks in the extrusion-based 3D printing of cell-laden hydrogel structures with high cellular viability [172]. K-carrageenan has been blended with Gel [173], with alginate to obtain a bioink with suitable structural strength and printability without affecting cell viability [174], with methylcellulose [175] to be used on tissue regeneration, or with another bioink for cell encapsulation and 3D bioprinting in tubular tissue regeneration [112]. Moreover, other works reported the addition of methacrylate groups to obtain a hydrogel with later UV (ultraviolet) crosslinking. This approach was used for encapsulating NIH-3T3 cells [176] to obtain a photocurable bioink through visible light for soft tissue injuries in situ healing [113] or to produce tissue scaffolds using DLP-based 3D printing technology [111].

Pullulan (PUL) is a non-ionic exopolysaccharide with a molecular weight ranging from 10 to 400 kDa, resulting in low-viscosity solutions when dissolved in water. To obtain hydrogels, PUL can undergo chemical modifications with methacrylate groups. This modification led PUL to the synthesis of a photo-crosslinkable hydrogel suitable for 3D printing while allowing spatial and structural control over the porosity and shape of the printed structure [177]. Additionally, this modification of PUL did not alter its rheologic properties but assured its printability in an extrusion 3D printing process. To the best of our knowledge, the use of pullulan for the preparation of cell-laden bioinks for 3D bioprinting has not been reported yet [20].

Pectin (Pc), a natural heteropolysaccharide extracted from plant cell walls, exhibits shear-thinning behavior that makes the extrusion process easier [178]. However, the low viscosity values of pectin solutions hinder their printability. For this reason, partial crosslinking through cation addition, either during the printing or in the post-printing process, is reported as a useful strategy to obtain table 3D structures [179,180]. Additionally, pectin can also be combined with other biopolymers such as oligochitosan [181] or Pluronic (Plu) to obtain scaffold structures crosslinked with Ca^2+^ [182] and containing microspheres with bioactive molecules, such as estradiol, for the generation of vascularized tissue [115]. Further, 3D bioinks based on high-methoxylated pectin with Manuka honey have been developed to produce wound dressings [183]. However, Pc has been primarily used in the enhancement of bioinks by adding bioactivity, among other important biochemical properties. This enhanced capacity has been reported in bioprinted cell-laden devices made of an alginate-pluronic hydrogel that supports the viability of pancreatic β-cells while adding immunomodulating capacity [114] or as a Gel thickening agent and promoter of bioprintability [184,185].

Despite different works reporting the application of protein or polysaccharide-based bioink, the blended between them arises as an excellent option since it is possible to meet the bioactive and structural properties of proteins and polysaccharides, respectively, as listed in Table 3.

## 3. Biopolymers Requirements for Bioprinting

**Figure 1:**
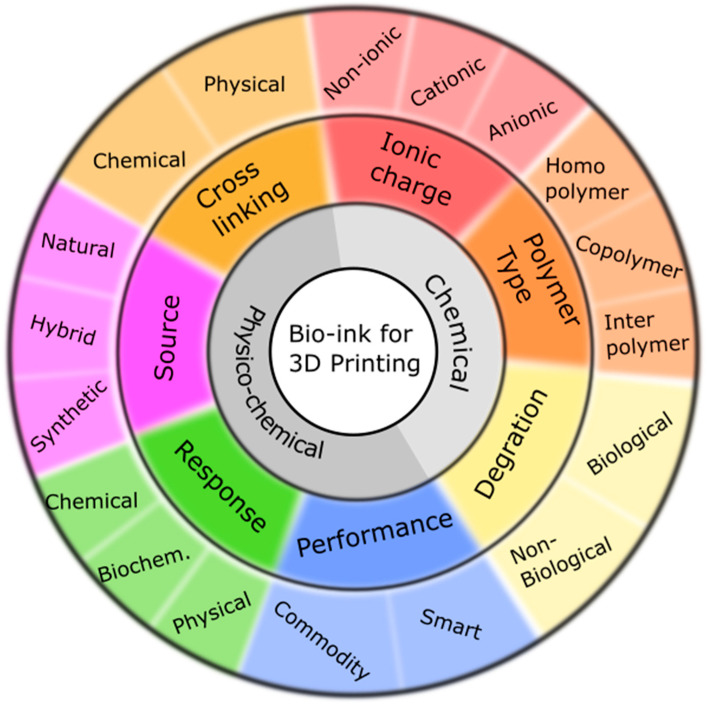
Classification of bioinks according to their properties.

In bioprinting, bioink is the term used to refer to a solution composed of natural and/or synthetic polymers selected for their good biocompatibility and rheological properties. Typically, bioinks are cell-laden gels with specific properties designed to be used in biofabrication (Figure 1). The two main components of these inks are biopolymers and solvents. So, the fundamental function of biopolymers is to maintain the most favorable conditions for the cells during the formulation and processing stages, as well as to act as a safe supporting substrate for the cells. Meanwhile, solvents’ main function is to provide appropriate rheological behavior and an adequate cell environment during the printing process. The concentration and the molecular weight of the biopolymer are critical in determining hydrogel viscosity behavior. Taking this into account, the most stable bioink formulations in the field of bioprinting are those using water as a solvent.

The combination of components and their proportion within the bioink formulation play a crucial role in establishing the printing conditions (e.g., pressure or temperature) and the overall quality of the printing outcome. For extrusion-based bioprinting, a high biomaterial concentration implies high viscosity, which usually has a negative impact on cell growth, migration, and differentiation, although it provides better fidelity control over the extruded material. Hydrogels with high molecular weight and low concentration biopolymers are commonly used and specifically formulated to determine the viscosity behavior, solubility shear rate, and working temperature of these hydrogels.

It is important to note that during the bioprinting process, bioinks undergo inner forces (compression and shear forces) that generate stress on the materials. The inner forces generated by each bioprinting technique must be considered during the setting because they have a major role in the bioink viscosity behavior, which controls important outcomes such as printability, fidelity, and cellular viability. This implies that bioinks must flow properly while maintaining their structural integrity to minimize cell stress and maintain the shape fidelity of the bioprinted structures.

The consolidation of the bioink is a crucial process that affects the qualities of the printed object and its application. The consolidation process determines the printing or bioprinting method to be used. During the consolidation process, the bioink undergoes a transition from its solvated (sol) state to its highly hydrophilic 3D reticulated macromolecule (Gel) state. The term “gelation” or “sol-gel transition” refers to this change of state. A polymer undergoes a transformation from its liquid or solvated state, exhibiting a pseudoplastic behavior (Sol), to a solid viscoelastic state (Gel), during the gelation process. In the case of bioinks, this consolidation process (sol-gel transition) is the result of the reactions of different macromolecular chains to gelate the bioink (crosslinking). The polymer’s size increases as a result of the macromolecular chain combination, eventually forming a stable or pseudo-stable three-dimensional network.

Depending on the crosslinking process, hydrogels can be classified into those that crosslink by chemical (permanent) or physical (typically reversible) bonds. The former creates a stable covalent chemical bond, while the latter relies on non-permanent physical processes, as crosslinking occurs via dynamic and reversible non-covalent (hydrophilic, electrostatic, or hydrogen) bonding interactions. Due to the rapid formation rate, physical crosslinking is the most interesting method to maintain the shape of the printed material.

The bioink structure should be reticulated to promote the adhesion and growth of loaded cells and reconstruct tissue-specific structures. The bioink can form crosslinked structures that contribute to the mechanical effects on cells, increasing both mechanical strength and biological activity. Physical crosslinking can be achieved using light and/or heat under specific conditions, while chemical crosslinking can be achieved by the addition of chemical or natural crosslinking agents. Cell survival is directly impacted by the stiffness of the print substrate. The mechanical and rheological properties of the bioink can be adjusted by modifying its composition. So, the bioink components should facilitate cell attachment, growth, and proliferation inside the 3D construct. At the same time, components should facilitate the modification of the biopolymers’ functional groups, promoting the inclusion and delivery of different biochemical signals or biomolecules.

It is common to apply a final fixing step or re-gelation process to maintain the final shape of the bioprinted object and keep a compromise between the different properties of the bioinks. Typically, this re-gelation stage is performed by incubating the bioprinted hydrogel in a solution with a physical or chemical crosslinking agent. Some common strategies utilized in bioprinting include adding photopolymerizing agents or agents that increase post-printing viscosity, facilitating crosslinking during these subsequent treatments. Another strategy is the addition of thermoplastic materials to mechanically reinforce and stabilize the structure of these printed objects [179,203,204,205].

## 4. Crosslinking of Biopolymers

The long-term stability and shape retention of a structure after printing are critical properties in bioprinting. The mechanism used by a hydrogel to achieve this is decisive when it comes to its use as a bioink. As previously mentioned, there are two types of crosslinking processes: chemical, which requires a crosslinking agent, and physical, which occurs through physical interactions between the biopolymer chemical groups and/or chains. Chemical crosslinking involves a molecule, the crosslinker, that reacts directly with the biomaterial and forms permanent interpolymeric bonds. Physical interactions are formed through various means, including electrostatic or macromolecule restructuration-type interactions, hydrogen bonds, the formation of stereogenic complexes, interpolymeric amphiphilic interactions, and reversible crystallization. Due to the lack of crosslinking agents, reversible hydrogels pose less toxicity, making them highly attractive for the development of bioinks [179,203,205,206,207,208,209].

### 4.1. Physical Crosslinking

Physically crosslinked polymers (Table 4) do not form any covalent bonds between chains, which implies that the bonds are formed through weak interactions. These bonds can be cyclically undone and re-done. Such behavior is typically seen in macromolecules with a specific preferred orientation and packaging, demonstrating a certain degree of order. Furthermore, these hydrogels have polar chemical groups that allow weak interactions, which can be broken with external stimuli such as temperature, pH, or similar, while exhibiting fast formation kinetics. Consequently, they are of great interest in the pharmaceutics and biomedicine fields, as they are not needed as crosslinking agents, which can be toxic/cytotoxic. Some of its counterparts have difficulty controlling the hydrogels’ physical properties, gelation time, network pore size, chemical functionalities, and degradation time. Moreover, enhancing their mechanical properties is challenging [207].

#### 4.1.1. Crystallization

The process of polymer crystallization consists of the formation of domains within the polymer matrix where different polymer sections are grouped together, increasing their arrangement, and clustering into pseudocrystalline structures (spherulites). This process affects the entire material, and multiple chain sections are involved in the formation of each domain. Therefore, the pseudocrystals reduce chain mobility and serve as crosslinking anchor points between consecutive chains. Polyvinyl alcohol (PVA) is one of the first hydrogels obtained using this type of crosslinking.

Polyvinyl alcohol (PVA) hydrogels, crosslinked by recrystallization, are gradually generated from an aqueous PVA solution at room temperature. The mechanical strength of these hydrogels manifests a significant dependence on the proceeding conditions. The molecular weight and the initial concentration are among those factors that influence the mechanical properties significantly. Additionally, the thermal history of the process and the gelation temperature have a significant impact. For example, the production of stable PVA hydrogel by crystallization at 37 °C may take up to 6 months. Hydrogels with this kind of crosslinking composition (PVA/CS, PVA/Starch, and PVA/Gel) are currently of interest in the fields of protein and peptide biotechnology as well as tissue engineering. Currently, there is a growing interest in the use of hydrogels with this crosslinking method in the fields of TE, protein, and peptide biotechnology. This includes the production of hydrogels with low-molecular-weight dextrin via recrystallization [203,205,206,207,208].

#### 4.1.2. Stereocomplex Formation

Stereocomplexation is a process wherein the recrystallization process of optical isomeric polymers of racemic blends undergoes recrystallization. Complexation is a consequence of the interactions generated by the stereoselective van der Waals forces. So, hydrogels crosslinked via this method show improved thermal and mechanical properties compared to their homocrystal counterparts. However, it is important to take into consideration the limited number of polymers compatible with this crosslinking method, such as mixtures of high molecular weight such as PDLA or PLLA. This mixture exhibits a phase transition or melting temperature (Tm) at higher temperatures (230 °C). In this sense, the modification of the melting temperature is attributed to the formation of stereocomplexes. Additionally, protein-based hydrogels are often formed from dextran-oligolactate suspensions. The degradation kinetics of these hydrogels are highly dependent on the number, length, and polydispersity of the lactate grafts, as well as the initial water content [179,203,205,207,208,210].

#### 4.1.3. Heating/Cooling

Heating/cooling crosslinked gels are generated through the processes of macromolecular chain recombination and conformational change, such as the restructuring of alpha-helix sections. For polymer solution temperatures above the melting point (Tm), the conformation of the macromolecular helix is lost. Posterior cooling redoes the helix structure due to intermolecular forces. This process involves the association of different macromolecule structures, leading to the formation of stronger junction zones, such as double helixes. Furthermore, when K^+^ or Na^+^ cations are present, the double helices further aggregate, forming more stable gels [203,207,210].

#### 4.1.4. Hydrogen Bonding

Hydrogen bond crosslinking is caused by the interaction between an electron-deficient hydrogen atom in a molecule and another functional group possessing a higher electron density in a closer molecule. In hydrogen bonds, the presence of protonated carboxylic acid groups is prevalent, and thus, the swelling of these gels is dependent on the environmental pH. This crosslinking process is usually found on gelatin-based hydrogels, such as blends of gelatin-agar, starch-carboxymethyl cellulose, or hyaluronic acid-methacrylate (HAMA). It is noteworthy that the hydrogel crosslinking process can be reversed with temperature when this hydrogen bridge formation does not happen at the same time as a macromolecular restructuring. Furthermore, other studies have explored hydrogel systems in which crosslinking is a result of the hybridization of hydrogen bonding and the stacking of bases [203,205,206,207,208].

#### 4.1.5. Ionic Interaction

One of the most widespread gelation processes is ionic interaction, which can be achieved under regular conditions such as room temperature and physiological pH. Usually, hydrogels based on ionic crosslinking are polysaccharide-based, with alginate-based hydrogels being the most commonly used. The crosslinking of alginate with polycationic solutions (Ca^2+^, Fe^2+^, and Fe^3+^) is feasible due to the presence of mannuronic and glucuronic acids. These gels are frequently used as matrices to encapsulate living cells and for protein delivery. Similarly, hydrogels can be produced by crosslinking CS, which contains 1,4-linked-β-glucosamine units, with glycerol-phosphate disodium salt. CS solutions below room temperature remain in a liquid phase when this salt is present but solidify quickly upon heating. Therefore, hydrogels that are in liquid form at room temperature but solidify at physiological temperature can be easily formulated. These gels can deliver active proteins, previously included in the hydrogel formulation before the gelation process, that stimulate bone and cartilage formation.

The addition of ions to produce hydrogels via ionic crosslinking does not require the presence of ionic groups in the polymer. Carrageenan is a polysaccharide containing 1,4-α-D-galactose and 1,3-β-D-galactose, as well as a few sulphate groups on its polymeric chain, that can form a gel either with potassium ions or under salt-free conditions. Furthermore, very rigid hydrogels can be produced in the presence of metallic cations. Dextran is another biopolymer that forms a hydrogel in the presence of potassium ions by encasing the potassium ions within the macromolecule structure. However, this hydrogel is not suitable for drug delivery due to its instability when in contact with water. Hydrogels can also be produced by the complexation of polyanions with polycations. The most frequently used ionically crosslinked CS hydrogels are the result of the complexation between CS and polyanions, such as dextran sulphate or polyphosphoric acid [203,205,207,208,209].

#### 4.1.6. Hydrophobicity

Hydrogels with a high water content, typically around 80 wt%, are physically crosslinked by hydrophobic interaction. This is achieved using compounds such as hydrophobic modified CS, dextran, pullulan, and carboxymethyl curdlan, which interact in the presence of water. These specific compounds are utilized in the formation of monodisperse hydrogel nanoparticles as a means of creating a microenvironment to safeguard proteins from thermal denaturation, aggregation, and enzymatic degradation. Furthermore, highly porous and low-swelling solid materials can be obtained via freeze drying and subsequent hydration with an alkaline buffer. This method can be used to obtain precise control over material properties and structure, producing materials that can increase their weight about 20 times without swelling [205,208].

#### 4.1.7. Maturation

The process of maturation consists of an increase in molecular weight, resulting in the production of a precisely structured hydrogel with controlled, structured molecular dimensions. An example of heat-induced gelation is the thermal treatment of gum Arabic, which contains about 2–3% protein [arabinogalactan protein (AGP), arabinogalactan (AG), and glycoprotein (GP)] as an integral part of its structure. So, these proteins are responsible for aggregation, leading to the improvement of their mechanical properties and water-binding capability [203,205].

### 4.2. Chemical Crosslinking

The formation of chemically crosslinked hydrogels, as represented in Table 5, is the result of a reaction between multifunctional monomers or groups and the biopolymer. The resulting hydrogel becomes insoluble in any solvent because of the formation of covalent bonds, which remain intact until breakage. Compared to physically crosslinked hydrogels, these hydrogels show better mechanical stability, resistance, longer degradation times, and the ability to regulate hydrogel formation. A wide range of chemical processes are available to form these hydrogels, such as radical polymerization, Michael reactions, Schiff based reactions, and enzymatic crosslinking reactions, based on the functional groups present (such as OH, COOH, NH_2_, and others).

#### 4.2.1. Complementary Groups

Chemical groups in hydrophilic polymers comprise atoms with varying electronegativities. As a result, some of these chemical groups, such as COOH, will be electron-deficient, while others, such as NH_2_ or OH, will be electron-dense. The presence of these groups in the polymer enables the creation of hydrogels through their reactivity, such as in the cases of amino-carboxylic acid, isocyanate-OH/NH_2_, or the synthesis of Schiff bases. Covalent bonds are created between the polymer chains during this process. A bifunctional molecule with the appropriate chemical group may be necessary as a crosslinking agent under certain circumstances. The dilution of the polymer’s aqueous starting solution will start hydrogel production. On the other hand, resins will be produced if the concentration is very high.

- Aldehyde, dihydrazide, and Schiff’s base

Aldehydes and dihydrazides (such as glutaraldehyde and adipic acid dihydrazide) are two important crosslinking agents. Crosslinking usually begins with low pH, high temperature, and methanol addition as a quencher. When preparing hydrogels, the polymer’s amino group can react with the aldehyde to form a Schiff base. The resulting imine link can be hydrolyzed, resulting in the breakdown of Schiff bases at low pH levels. So, using dialdehyde or formaldehyde as a crosslinking agent makes it possible to form gelled hydrogels based on proteins such as Gel, albumin, and amine-containing polysaccharides. In this sense, some hydrogel films are made by modifying HA with adipic dihydrazide and then crosslinked with poly (ethylene glycol)-propiondialdehyde. These hydrogels are commonly used for controlled medication delivery [210].

- Thiol-ene Michael addition

The thiol-based Michael-type reaction has been investigated as a viable method for producing chemically crosslinked hydrogels due to its mild reaction conditions, well-characterized reaction processes, and capacity to create homogeneous constructions. This chemical crosslinking is based on nucleophilic addition to unsaturated carbonyl compounds. Typical nucleophiles include thiol and amine-bearing molecules, while unsaturated carbonyl can be found in acrylate, methacrylate, and vinyl sulfone groups. Photoinitiated thiol-ene crosslinking has recently been investigated as a desirable method for cell encapsulation. This photopolymerization mode requires highly reactive components and a short exposure time. So, cells can benefit from lower exposure to reactive radicals. Additionally, the thiol-ene chemistry has a reduced susceptibility to oxygen inhibition, allowing cell encapsulation in an oxygen-rich environment, which is a desirable attribute for 3D bioprinting technologies [210].

- Condensation

Condensation reactions are an effective method for crosslinking water-soluble polymers. Therefore, their application in hydrogel creation is being encouraged. The Passerini and Ugi condensation processes are extensively used in the synthesis of polymers, specifically in the production of polyesters and polyamides. For this reason, biopolymers that present hydroxyl groups or amines with carboxylic acids or derivatives are necessary, respectively. To facilitate the sol-gel transition, the crosslinking reaction can be conducted in slightly acidic water at room temperature. A few molecules are commonly included in this process to prevent any unwanted side reactions and to achieve tighter control of the gel’s crosslinking density. The rate of gel degradation at room temperature and pH 9.5 can be adjusted based on the crosslinking density. The inclusion of bioactive molecules, such as antimicrobial proteins, lysozymes, or negatively charged polysaccharides, to increase loading capacity is another intriguing alteration [205,207,208,211].

#### 4.2.2. Radical Polymerization

Radical polymerization has become the most widely used technique for creating chemically crosslinked hydrogels. Specifically, two reaction subclasses can be further classified under this category: random and controlled polymerization. To produce hydrogels through free radical processes, a monomer, oligomer, or macromer with unsaturated vinyl functional groups must undergo polymerization. The macromolecular backbones are activated in chemical grafting via radical polymerization, which occurs through the reaction of a chemical reagent (visible or UV light, heat, or redox initiators). This crosslinking technique is highly effective and frequently utilized, even in modest conditions. The characteristics of the hydrogel and its swelling capacity depend on the proportion of crosslinker used. The most typical natural polymers used in radical crosslinking are HA, dextran, and CS, as well as biopolymers functionalized with acrylate groups. Therefore, photocrosslinking techniques are typically the most promising for using cell-laden bioinks in bioprinting [205,207,208,209,211].

Additionally, the classification of a radical initiation method can be extended to include thermal and photoinitiation processes, as well as polymerization induced by heat and/or light. This crosslinking by light-induced polymerization is an incredibly quick hydrogel process that is particularly attractive for the creation of patterned structures. The selection of the appropriate photoinitiator type and solvent is crucial, as they may leak out of the hydrogel post-production. The radicals produced during polymerization in the presence of proteins can harm the protein’s structure. Additionally, the UV light’s intensity is restricted to prevent cell damage because the heat released during the crosslinking process may result in cell death [179,205,207].

#### 4.2.3. Enzymatic

Enzymatic crosslinking offers excellent substrate selectivity and avoids adverse responses. The peptide ratio can be changed to customize the gel’s properties. The reaction kinetics of the gel are influenced by the ratio of reactants, enzyme concentration, and macromer shape and composition. This qualifies these hydrogels as suitable for in situ gelling systems.

Transglutaminase (TG), a Ca^2+^-dependent enzyme, is capable of catalyzing the formation of polypeptide-like hydrogels rapidly at biological temperature (37 °C). The TG forms these hydrogels by amide linkage between the carboxamide and primary amines on polymers or polypeptides. Horseradish peroxidase and H_2_O_2_ have been used in similar proceedings to crosslink hydrogels based on HA, dextran, cellulose, and alginate [180,204,205,206,210,211].

## 5. Printing Techniques for Biopolymers

Bioprinting is defined by the Cambridge English Dictionary as ‘the process of producing tissue or organs similar to natural body parts and containing living cells using 3D printing’. The field of bioprinting has experienced tremendous growth over the last decade as it allows the deposition of biomaterials with cells following precise geometries that aim to mimic the tissue or organ structures present in the body. One of the current and potential uses of bioprinting is the fabrication of viable tissues and organs for application in regenerative medicine, the creation of biomimetic in vitro models for drug screening, and the use of bioprinting in personalized medicine through a collection of tissues obtained from the same patients receiving the treatment.

The cells used in bioprinting require an environment capable of providing, at the same time, adequate mechanical support for shape retention and a biocompatible environment to allow cell growth while maintaining their functionality as closely to their native physiological environment as possible. For this purpose, printing typically follows the structure of porous scaffolds, with pores providing an area for cell growth and nutrients and oxygen penetration within the printed construct. Natural polymers, as opposed to synthetic ones, offer advantages in terms of better biocompatibility, biodegradability, and mimicking of the physiological properties of extracellular matrix (ECM) [212] e.g., stiffness.

**Figure 2:**
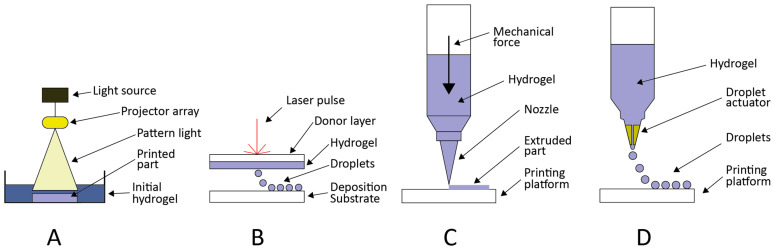
Different printing techniques used with biopolymers: (**A**) photopolymerization, (**B**) laser, (**C**) extrusion and (**D**) droplet.

Different biopolymers and gelation techniques have been used to generate bioinks. Therefore, printing techniques have been developed based on different principles, printing parameters (Table 6), and foundations adapted to each hydrogel and cell type. In this sense, these printing technologies can be divided into photopolymerization, laser-assisted, and deposition printing approaches depending on the underlying concepts upon which they are founded (Figure 2). Therefore, the properties of these scaffolds and their ultimate application hinge on the manufacturing procedure used to create them.

### 5.1. Photopolymerization-Based

In photopolymerization-based printing, resins contain a photoinitiator activated by laser to produce the photopolymerization of a small volume around the focus spot. For this reason, the selection of an adequate photoinitiator agent is critical for biocompatibility since some have cytotoxic effects [222]. Furthermore, the photoinitiator will affect the final resolution, and occasionally a photoinitiator and a photoblocker are combined to achieve a smaller spot size.

Several techniques using photopolymerization principles have been developed, including stereolithography (SLA), digital light printing (DLP), and two-photon polymerization (2PP).

SLA works by using a mirror (galvanometer) to focus a laser beam on the desired spot, typically inside a resin tank, and achieves high resolution at a relatively low printing speed. Each layer of the object is printed by scanning dot by dot with the mirror (Figure 2A). Improvements in SLA technology have led to DLP, a more efficient method in which a digital light projector replaces the laser beam, curing each full layer at a time. Resolution in SLA and DLP printers is typically within 10–75 μm and rapidly improving. 2PP is based on photopolymer crosslinking induced by high-intensity femtosecond laser pulses. Due to the non-linear nature of the two-photon absorption, proportional to the square of the light intensity, it is possible to achieve very small voxel sizes, leading to submicrometer resolution [212], albeit with longer printing times.

The range of materials available for photopolymerization was initially restricted to photocurable synthetic resins, including hydrogels made from modified biopolymers. In this sense, it should be noted that the materials used in photopolymerization are not strictly natural polymers but modified polymers including acrylate or diacrylate groups, allowing the polymerization process to occur after the photoinitiator has been excited. Vinyl chemical groups are often incorporated through modification with acrylates, such as methacrylic anhydrides. Thus, polymers such as gelatin methacrylate (GelMA), hyaluronic acid methacrylate (HAMA), or Col methacrylate (ColMA) are among those commonly used.

The biocompatibility of constructs produced by photopolymerization may be limited by several factors. Photoinitiators are required in the hydrogel, which could harm biocompatibility through possible residual toxic photocuring agents [222]. Additionally, UV and shorter wavelengths of light may cause mutations in DNA, and intense light can decrease cell viability. Therefore, caution is needed while selecting and utilizing photoinitiators, as well as the wavelength and intensity of the light. Printing time is another crucial consideration because long print durations will result in insufficient immersion of cells in the medium and a lack of necessary CO_2_ supply and humidity [214]. The porosity of the printing scaffold and the rheological properties of the hydrogel will determine whether the cells can obtain enough oxygen and nutrients, further spread, and migrate through the material, which is another important consideration.

Postprocessing in photopolymerization bioprinting affects both the structural properties of the construct and cell viability. Typically, these samples need to be immersed in a bath with a solvent (e.g., water or isopropanol) that removes the non-crosslinked volume, and in some cases, the extra support material must be mechanically removed. Furthermore, inks made of synthetic polymers commonly contain organic solvents that are usually cytotoxic. Thus, post-printing cell seeding is more prevalent than cell-laden photopolymerization-based bioprinting.

Examples of photopolymerization-based bioprinting are numerous. Both SLA and DLP have been used to produce vascular grafts and vascularized tissue constructs [213]. These techniques allow the building of cylindrical structures with a very narrow lumen due to their high resolution. For the same reason, they are also used for printing other tubule-like structures, such as nerve conduits [223,224]. 2PP lithography was initially limited to resin-like materials [225], resulting in biomimetic models that are particularly useful for tissue engineering, such as trabecular bone [226,227], cardiac tissue [200], or blood-brain barrier [228]. Nowadays, natural polymers find application in various fields, such as printing microvasculature channels in Col hydrogels [229,230], creating structures for cell encapsulation in GelMA [231], or reproducing the microstructure of a lung’s alveolar tissue parenchyma in gelatin methacryloyl-based resin [232].

### 5.2. Laser-Based

In laser-assisted bioprinting (LAB), a laser beam is focused on the upper surface of a bioink layer affixed to a transparent support. In response to the laser, a bubble forms at the focus spot, making the ink stream along the vacuolar membrane. After the bubble collapses, the ink forms a jet or droplet that is deposited on the substrate [220], which typically contains a biopolymer or cell culture medium to ensure cell adhesion (Figure 2B) [216]. The small size of the droplets guarantees a higher resolution than extrusion-based bioprinting. Despite the numerous factors potentially affecting cell viability in this technique (laser radiation, thermal changes, mechanical forces during the transfer and landing processes), LAB has been reported to achieve viabilities of over 90% [215,217]. LAB allows printing using more viscous bioinks than extrusion-based systems, as the ink does not pass through a nozzle [220].

LAB has been used to print cellularized skin grafts in a mouse skin model using fibroblasts and keratinocytes positioned in a Col solution on the surface of a commercially available Col/elastin matrix (Matriderm^®^) [116]. After 11 days of implantation, the grafts were explanted and analyzed, revealing that the printed cells had formed a tissue similar to the native mice skin. This formation included a dense epidermis and the formation of a microvascularization network.

### 5.3. Extrusion-Based

In this bioprinting technique, hydrogels are extruded through a nozzle or printing head and deposited over a substrate to construct layer-by-layer through a mechanical force (Figure 2C). Due to its working mechanism, the bioink must have adequate rheological properties, provide good extrudability, and maintain structural integrity after printing. These requirements, often referred to as printability, contribute significantly to the regulation of cell functioning [233,234]. Thus, the design of a hydrogel for extrusion bioprinting must consider both rheological and biological factors. Bioinks are composed of one or more biomaterials mixed with living cells and eventually other biologically active components that impact their flow behavior. The viscosity of bioinks must be low enough to allow the extrusion process without requiring high pressure. This high pressure causes excessive shear stress, which can dramatically affect cell viability [235]. On the other hand, the retention of the shape of the construct after the extrusion process is primarily influenced by the material yield stress and, secondly, by its viscosity.

Crosslinking/gelation can also be of crucial importance in controlling both printability and cell function in extrusion-based bioprinting. Hydrogel gelation can be performed before, during, and after the extrusion printing procedure. So, pre-printing crosslinking can be used as a printability enhancer to obtain gel-like materials adequate for printing. This procedure must be carefully considered due to the possibility of provoking nozzle clogging, and thus, decreasing the material’s extrudability [234] or even inducing the total gelation of the bioink, which is then no longer extrudable. Additionally, crosslinking while the printing process is ongoing, such as immediate photopolymerization after the bioink exits the nozzle, helps in maintaining the structural integrity of the printed part. However, crosslinking agents activated by visible light are preferred compared to those activated by UV light, as they may damage cells, and the presence of photoinitiators may also have deleterious effects on the cells [223]. Finally, post-printing crosslinking is typically accomplished by a light source, thermal treatment, or the addition of a metal-ion source such as CaCl_2_. However, thermal post-printing treatment in cell-laden hydrogels is limited by the presence of the cells and the range of temperatures they can stand. The addition of CaCl_2_, which is generally well-tolerated by most cell types, could affect the survival of those cells strongly affected by the presence of X^2+^ ions, such as cardiomyocytes.

Alginate/gelatin (AlgGel) hydrogel is a widespread example of a natural polymeric bioink for extrusion bioprinting due to its excellent printability, biocompatibility, and biodegradability. Alginate is renowned for its printability properties, but it lacks biological activity, while Gel enhances biological activity, resulting in a biocompatible and biodegradable easy-to-print bioink [207]. This combination has been used, for example, to print cardiac patches, mixing the hydrogel with human coronary artery endothelial cells (HCAECs) and separately with mouse vascularized cardiac spheroids (VCSs) [236]. The results showed high viability at 28 days, with the HCAECs successfully establishing a vascular network and the VCSs showing contractility. Another example is the printing of bilayer renal tubular tissue with a decellularized kidney ECM mixed with alginate using a coaxial extruder [237].

### 5.4. Droplet-Based

In droplet-based bioprinting (DBB), the structure is built by small bioink droplets deposited on a substrate. There are different approaches to this technique: inkjet, acoustic, and microvalve bioprinting (Figure 2D) [238]. Similar to extrusion bioprinting, bioinks used in DBB require low viscosity, biocompatibility, and biodegradability.

Printing hydrogel droplets on a substrate is the most frequent approach, but an alternative approach involving printing droplets containing cells or other biologicals on top of hydrogel substrates has also been reported [238]. For instance, skin graft substitutes have been fabricated by printing a solution of human dermal microvascular endothelial cells mixed with thrombin onto a substrate made of Col type I with neonatal human fibroblasts [239]. These skin grafts were implanted in a mouse model, demonstrating improvements in wound contraction.

Besides Col, several natural polymeric hydrogels have been reported as effective options for DBB. Alginate is frequently used in DBB and is often preferred due to its excellent printability, for example, in printing microvasculature constructs [240]. GelMA is also one of the most commonly used natural polymer hydrogels due to its degradability and tunable mechanical properties. GelMA has been used to recreate tumor microenvironments by mixing co-cultured CAL27-CALF tumor spheroids with a GelMA-based bioink and printing them using acoustic droplet printing [241]. F has been used to produce microvasculature by inkjet printing droplets of thrombin loaded with human dermal microvascular endothelial cells on top of a fibrinogen substrate, forming a microcapillary network after 21 days of culture [241].

## 6. Discussion

3D bioprinting is a recent and promising technology for TE but still presents several challenges to solve. The most relevant ones are: (1) the identification of biodegradable and biomimetic printable biopolymers that enable prompt cell adhesion and proliferation; (2) the need for vascularization at the single-cell level; (3) the development of complex patterning of heterocellular tissues; and (4) the conservation of cell viability and long-term post-printing functionality until the remodeling and regeneration of the tissue are completed [179,206,209,228].

**Figure 3:**
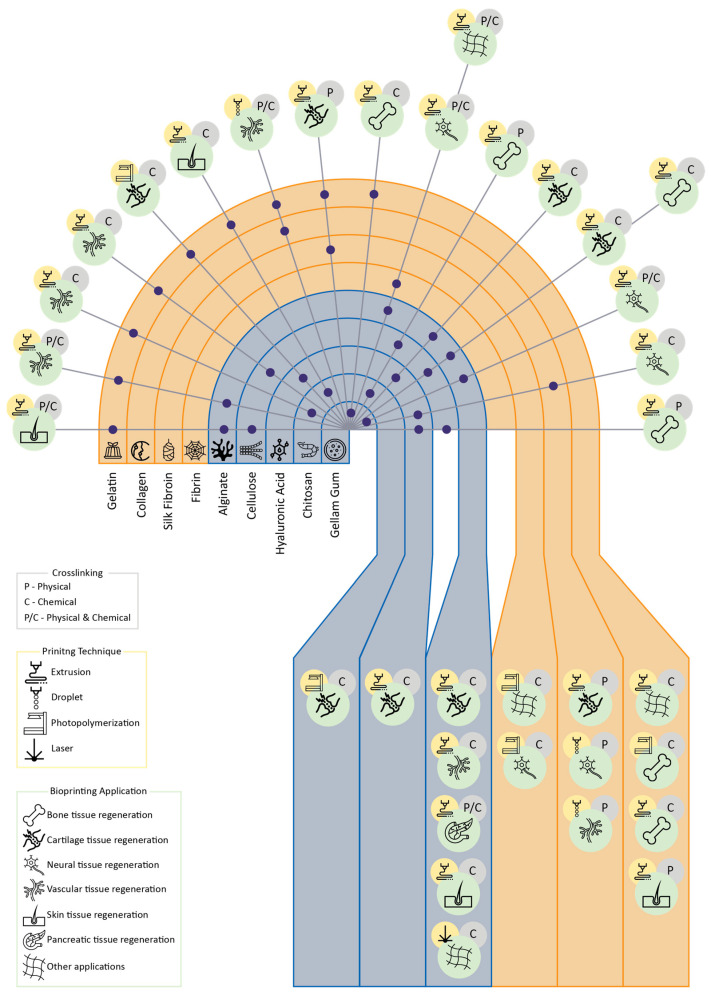
General overview of the data analysis where the most used biopolymers are classified with circles according to: (1) their application (green), (2) the printing procedure (yellow), and (3) the crosslinking type (grey). Hydrogel’s blend is represented in the upper part (purple dots), while one-biopolymer hydrogel appears in the lower part of the figure, although other materials can be part of the blend (not included for simplicity).

Figure 3 presents the most relevant data extracted in this review, showing that biopolymers are commonly used in six specific TE applications: bone, cartilage, neural, vascular, skin, and pancreatic tissue regeneration. Other applications are included under a general concept named other tissue regeneration. This figure represents the interaction of commonly used biopolymers, their bonding into new hydrogels, and the printing and crosslinking techniques used for each application.

## 7. Conclusions and Future Perspectives

In conclusion, hydrogels based on biological source compounds, namely, polysaccharides and proteins, are widely used in the composition of bioinks due to their biocompatibility, biodegradability, and resemblance to some properties of the ECM (e.g., stiffness). The potential of natural-based bioinks has been evaluated in several TE applications, such as bone, cartilage, neural, vascular, skin, and pancreatic tissue regeneration. These types of bioinks have been studied due to their biocompatibility, flexibility to be chemically modified or blended to improve their printability and mechanical stability during and after the printing process, and also to augment the biological or mechanical features of the 3D-printed structures. However, the rheological properties of natural compounds are unsuitable to be used alone as bioinks. So, several procedures to improve their printability are commonly used, such as blending with other natural or synthetic materials or chemical modification. In general, alginate and gelatin are biopolymers widely used in TE for a variety of applications, together with other methacrylated biopolymers (GelMA, ColMA, and HAMA). However, new alternative biopolymers are currently under study to avoid undesirable effects that compromise cell proliferation and maturation using Michael reactions for crosslinking.

Furthermore, future research may also attempt to optimize the printing techniques and printing process parameters to develop specific multifunctional bioprinted constructs. By combining state-of-the-art TE strategies and achievements made by current and ongoing research, it aims to propel the development of ideal 3D bioprinted materials based on biopolymers and consequently their successful translation into clinical applications in the future.

## Figures and Tables

**Table 1:** Protein-based bioinks used for tissue engineering purposes.

Bioink Compounds ^1^	Cells ^2^	Printing Techniques	Applications	Reference
Col	HCCs	Extrusion	Cartilage tissue regeneration	[31]
Col + VEGF	NSCs	Droplet	Neural tissue regeneration	[32]
Gel + PU	MSCs	Extrusion	Tissue regeneration	[33]
Gel + GelMA + OCP	HUVECs	SLA	Bone regeneration	[34]
Gel + GelMA	Endothelial and ASCs	Extrusion	Bone regeneration	[35]
SF + GMA	NIH/3T3 and chondrocytes	DLP	Heart, vessel, brain, trachea, and ear regeneration	[36]
SF_GMA + GO	Neuro2a	DLP	Neural tissue engineering	[37]

^1^ Biopolymer acronyms: Collagen (Col), Vascular Endothelial Growth Factor (VEGF), Gelatin (Gel), Polyurethane (PU), Methacrylate Gelatin (GelMA), Octacalcium Phosphate (OCP), Silk Fibroin (SF), Glycidyl Methacrylate (GMA), Glycidyl Methacrylate Silk Fibroin (SF_GMA), Graphene oxide (GO). ^2^ Cells acronyms: Human-derived Chondrocyte cells (HCCs), Neural Stem cells (NSCs), Mesenchymal Stem cells (MSCs), Human Umbilical Vein Endothelial cells (HUVECs), Adipose-derived Stem cells (ASCs), embryonic mouse fibroblast cells (NIH/3T3), and mouse neural crest-derived cells (Neuro2a).

**Table 2:** Polysaccharide-based bioinks used for tissue engineering purposes.

Bioink Compounds ^1^	Cells ^2^	Printing Techniques	Applications	Reference
Alg + Aga	FBCs	Extrusion	Cartilage tissue engineering	[105]
Col + Fg	HUVECs and HDFs	Droplet	Vascular tissue regeneration	[106]
CS + PEGDA	hMSCs	SLA	Cartilage tissue engineering	[107]
HA + ChS	MSCs	Extrusion	Cartilage tissue engineering	[108]
Gel + XG	Fibroblasts and keratinocytes	Extrusion	Skin regeneration	[109]
XG + GOx + Glu + NHS	NIH/3T3	Extrusion	Soft tissue engineering	[110]
MA-κ-CA	NIH/3T3	DLP	Soft tissue engineering	[111]
MA-κ-CA + Alg	HUVECs	Extrusion	Vascular tissue regeneration	[112]
MA-κ-CA	HeLa and Fibroblasts	Extrusion	Soft tissue engineering	[113]
Pc + Plu + Alg	MIN6	Extrusion	Pancreatic tissue engineering	[114]
Pc + Plu	mBMSCs	Extrusion	Vascular tissue regeneration	[115]
Alg	NIH/3T3 and hMSCs	Laser	Skin regeneration	[116]
Alg + Fg	AC16	Extrusion	Myocardial regeneration	[78]

^1^ Biopolymers acronyms: Alginate (Alg), Agarose (Aga), Collagen (Col), Fibrinogen (Fg), Chitosan (CS), Polyethylene Glycol Diacrylate (PEGDA), Hyaluronic Acid (HA), Chondroitin sulfate (ChS), Gelatin (Gel), Xanthan Gum (XG), Glucose Oxidase (GOx), Glucose (Glu), N-Hydroxy Sulfosuccinimide (NHS), Photocurable Methacrylate-κ-Carrageenan (MA-κ-CA), Pectin (Pc), Pluronic F127 (Plu). ^2^ Cells acronyms: Fetal Bovine Chondrocytes (FBCs), Human Umbilical Vein Endothelial cells (HUVECs), Human Dermal Fibroblasts (HDFs), Mesenchymal Stem cells (MSCs), Human Bone Marrow Mesenchymal Stem cells (hBMSCs), Embryonic Mouse Fibroblast cells (NIH/3T3), Mouse Insulinoma cells (MIN6), Mouse Bone Marrow Mesenchymal Stem cells (mBMSCs), AC16 cardiomyocytes (AC16).

**Table 3:** Protein- and Polysaccharide-blended bioinks used for tissue engineering purposes.

Bioink Compounds ^1^	Cells ^2^	Printing Techniques	Applications	Reference
F + Alg + G	U87MG	Extrusion	Tissue construct	[186]
Col + Alg + TH	Fibroblasts	Extrusion	Vascular tissue regeneration	[187]
Col + GelMA	HUVECs and hMSCs	Droplet	Vascular tissue regeneration	[188]
SF + Gel	hMSCs	Extrusion	Cartilage tissue engineering	[189]
F + Alg + G	ASCs	Extrusion	Neural tissue engineering	[190]
F + G + Alg	hiPSCs	Extrusion	Neural tissue engineering	[191]
Alg + Gel + DEAE-C + Fg	HPFs and keratinocytes	Extrusion	Skin tissue engineering	[192]
Alg + TOCNF + PDA-NPs	Osteoblasts	Extrusion	Bone tissue regeneration	[193]
Alg + Gel + Soy	HUVECs	Extrusion	Vascular tissue regeneration	[194]
aAlg + aHA	MSCs	Extrusion	Cartilage tissue engineering	[195]
tCS + GHEC + CNCs	MC3T3-E1	Extrusion	Bone tissue regeneration	[196]
sCS + aDex + GelMA	hBMSCs and HUVECs	Extrusion	Vascular tissue regeneration	[165]
HAMA + Col	PC-12		Neural tissue engineering	[197]
HA + CMC	MC3T3	Extrusion	Bone tissue regeneration	[198]
HAMA + GelMA	CCs	Extrusion	Cartilage tissue engineering	[199]
TOCNF + GelMA	ASCs	Extrusion	Vascular tissue engineering	[200]
TOCNF + Alg	hMF	Extrusion	Cartilage tissue engineering	[201]
GGMA + GelMA + DFO-Eth	HUVECs and hBMSCs	Extrusion	Bone regeneration	[159]
GG + Alg + LM	hiPSCs	Extrusion	3D neuromodeling	[202]
aDex + sCS + GelMA	hBMSCs and HUVECs	Extrusion	Skin regeneration	[165]

^1^ Biopolymers acronyms: Fibrin (F), Alginate (Alg), Genipin (G), Collagen (Col), Tyramine Hydrochloride (TH), Methacrylate Gelatin (GelMA), Silk Fibroin (SF), Gelatin (Gel), Diethylaminoethyl Cellulose (DEAE-C), Fibrinogen (Fg), TEMPO-oxidized Cellulose Nanofibril (TOCNF), Polydopamine Nanoparticles (PDA-NP), Gelatin (Gel), Aldehyde Alginate (aAlg), Amine-Hyaluronic Acid (aHA), Thermogelling Chitosan (tCS), Glycerophosphate Hydroxyethyl Cellulose (GHEC), Cellulose Nanocrystals (CNC), Succinylated Chitosan (sCS), Dextran Aldehyde (aDex), Methacrylated Hyaluronic Acid (HAMA), Hyaluronic Acid (HA), Carboxymethylcellulose (CMC), Methacrylated Gellan Gum (GGMA), Deferoxamine-loaded ethosomes (DFO-Eth), Gellan Gum (GG), Laminin (LM). ^2^ Cells acronyms: Glioblastoma cells U87MG (U87MG), Human Umbilical Vein Endothelial cells (HUVECs), Human Mesenchymal Stem cells (hMSCs), Adipose-derived Stem cells (ASCs), Human induced Pluripotent Stem cells (hiPSCs), Human Dermal Fibroblasts (HPFs), Mesenchymal Stem cells (MSCs), Pre-osteoblastic cells (MC3T3-E1), Human Bone Marrow Mesenchymal Stem cells (hBMSCs), Rat adrenal phaeochromocytoma cells (PC-12), Porcine Chondrocytes cells (CCs), Human Meniscus Fibrochondrocytes (hMF).

**Table 4:** Different kinds of physical crosslinking with some examples of biopolymers and their chemical schema.

Physical Crosslinking	Biopolymers	Schema	References
Crystallization	Chitosan/PVA Gelatin/PVA Strach/PVA Dextran	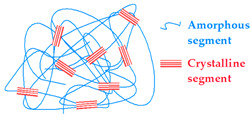	[203,205,206,207,208]
Stereo complexation	Dextran	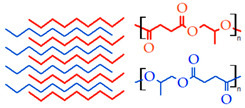	[179,203,205,207,208,210]
Heating/Cooling	Gellan Gum Gelatin Carrageenan	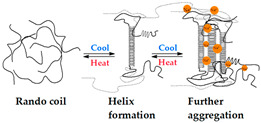	[203,207,210]
Hydrogen bonding	Gelatin/Agar Starch/Carboxymethyl Cellulose Hyaluronic Acid/Methyl Cellulose	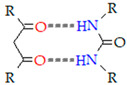	[203,205,206,207,208]
Ionic interaction	Alginate Chitosan Carrageenan	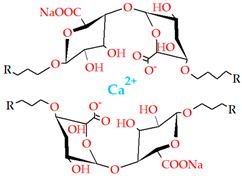	[203,205,207,208,209]
Hydrophobicity	Chitosan Dextran Pullulan Carboxymethyl curdlan	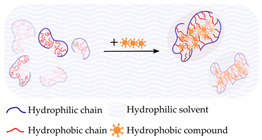	[205,208]
Maturation	Alginate Chitosan Carrageenan Arabic gum	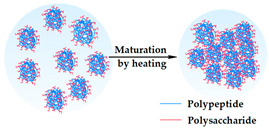	[203,205]

**Table 5:** Different kinds of chemical crosslinking, with some examples of biopolymers and their chemical schema.

Chemical Crosslinking	Biopolymers	Schema	Reference
Complementary Groups			
1. Aldehyde, Dihydrazide, and Schiff’s base	Hyaluronic acid-based Dextran Chitosan Alginate	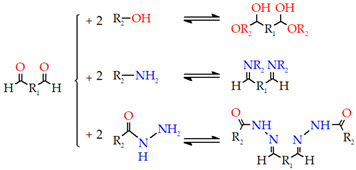	[210]
2. Thiol-ene Michael addition	Hyaluronic acid-based Dextran Chitosan Alginate	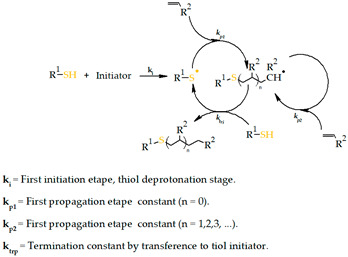	[210]
3. Condensation	Hyaluronic acid-based Dextran Chitosan Alginate	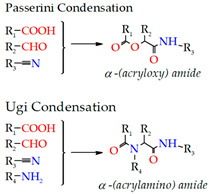	[205,207,208,211]
Radical polymerization	Hyaluronic acid Dextran Chitosan	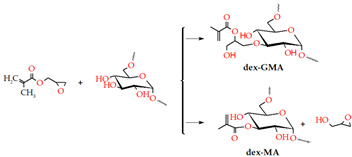	[205,207,208,209,211]
Enzymatic	Gellan gum Polypeptides Hyaluronic acid Dextran Cellulose Alginate	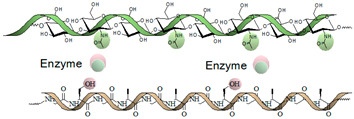	[180,204,205,206,210,211]

**Table 6:** Bioprinting techniques and their printing parameters.

Bioprinting Technique	Parameters	Reference
Photopolymerization	Wavelength Light/laser power Exposure time Repetition rate Pulse width Environmental temperature	[212,213,214]
Laser-based	Wavelength Laser intensity/power Pulse rate/frequency Laser fluence Hydrogel viscosity Thickness of the absorbing layer Thickness of the bioink layer Travel distance Printing velocity	[215,216,217]
Extrusion	Printing pressure Printing velocity Nozzle height Flow rate Printing temperature Hydrogel viscosity Extrusion gauge Environmental temperature	[217,218,219]
Droplet	Pressure Droplet rate Droplet volume Hydrogel viscosity Kinetic momentum Printing speed Printing time Voltage pulse (amplitude, rise and fall times, dwell time, echo time, and frequency) Substrate hydrophobicity/hydrophilicity	[212,220,221]

## Data Availability

No new data was created or analyzed in this study. Data sharing is not applicable to this article.
